# Olfactory Dysfunction in COVID-19 Patients Who Do Not Report Olfactory Symptoms: A Pilot Study with Some Suggestions for Dentists

**DOI:** 10.3390/ijerph19031036

**Published:** 2022-01-18

**Authors:** Riccardo Favero, Silva Hajrulla, Anna Bordin, Carla Mucignat-Caretta, Piergiorgio Gaudioso, Bruno Scarpa, Lorenzo Favero, Giancarlo Ottaviano

**Affiliations:** 1 Dentistry Section, Department of Neurosciences, University of Padua, 35128 Padua, Italy; rickyfavero@msn.com (R.F.); silva.hajrulla@studenti.unipd.it (S.H.); lorenzo.favero@unipd.it (L.F.); 2 Section of Otolaryngology, Department of Neurosciences, University of Padua, 35128 Padua, Italy; anna.bordin.pd@gmail.com (A.B.); piergiorgio.gaudioso@studenti.unipd.it (P.G.); 3 Department of Molecular Medicine, University of Padua, 35128 Padua, Italy; carla.mucignat@unipd.it; 4 Department of Statistical Sciences, University of Padua, 35121 Padua, Italy; bruno.scarpa@unipd.it; 5 Department of Mathematics “Tullio Levi Civita”, University of Padua, 35121 Padua, Italy

**Keywords:** COVID-19, smell, olfactory disfunction, dentistry

## Abstract

Background: Smell and taste dysfunction are frequently reported by SARS-CoV-2 positive patients. The degree of olfactory and gustatory dysfunction varies from a very mild reduction to their complete loss. Several studies have been performed to determine their prevalence in COVID-19 patients, mostly using subjective measurement methods. The literature lacks long-term studies regarding duration and recovery. Methods: We assessed olfactory performance, using the Sniffin’ Sticks olfactory test, in a group of patients who had not reported olfactory dysfunction, around 131 days after their COVID-19 diagnosis. Results: 11 out of 20 subjects showed no olfactory reduction (65%), while 9 subjects showed reduced TDI score (45%). A total of 13 subjects (65%) scored above the cutoff point for Threshold, 16 subjects (80%) scored above the cutoff point for discrimination and 13 subjects (65%) scored above the cutoff point for identification. Conclusion: Objective measurement methods of olfactory performance show a higher prevalence of olfactory reduction compared to patients’ self-reported questionnaires. Olfactory dysfunction can last even months after its onset and because of its high prevalence, it could be a screening symptom for suspect COVID-19 cases.

## 1. Introduction

The COVID-19 outbreak started in Wuhan, China, where several unusual cases of pneumonia were reported at the end of 2019. Clinical symptoms were mainly represented by cough, dyspnea, fever, myalgia, fatigue and even acute respiratory distress syndrome (ARDS) [1]. COVID-19 is caused by SARS-CoV-2, a virus that is part of the coronaviridae family, just like SARS-CoV (Severe Acute Respiratory Syndrome Coronavirus, 2003) and MERS-Cov (Middle East Respiratory Syndrome Coronavirus, 2012) [2]. The Coronaviridae family is divided into alpha, beta, gamma and delta coronaviruses; beta coronaviruses can infect humans. Generally, coronavirus infections cause mild diseases in humans with symptoms similar to the common cold, but some of these viruses, like SARS-CoV, MERS-Cov and SARS-CoV-2 have proved to be a real threat to people’s health and may cause death. SARS-CoV-2 is a beta coronavirus composed of a single-stranded RNA, surrounded by a lipid bilayer and membrane proteins. It enters the human body by binding the ACE-2 receptor, which is widely spread in the respiratory system [3].

Due to the contagiousness of the virus, the number of cases increased rapidly all around the world and the World Health Organization declared the COVID-19 outbreak a global pandemic on 11 March 2020 [4,5]. Ever since the outbreak of the infection, in addition to pneumonia, several symptoms have been associated with COVID-19. While some patients display relevant symptoms, others are completely asymptomatic or might present mild symptoms (paucisymptomatic). The virus has an incubation period that ranges from 2–14 days and is most likely to be transmitted from infected to healthy individuals around the fifth day of illness [3]. Asymptomatic subjects can also be responsible for secondary infections, although at a lower degree compared to symptomatic subjects [3]. Screening procedures put in place to reduce contacts between healthy and infected people and limit transmission, often fail to recognize asymptomatic and pre-symptomatic individuals and also individuals that present very mild symptoms (sub-clinical cases) [6]. Studies have shown that children present milder symptoms compared to adults; nonetheless, they are potential carriers and may transmit the infection [7].

Several studies report central and peripheral nervous system involvement, which manifests with symptoms such as headache, dizziness, delirium, anosmia and dysgeusia, oculomotor impairment, etc. [8].

Olfactory and gustatory dysfunction generally appear around the third or fourth day after the onset of symptoms; in some cases, they even precede other symptoms [9,10]. Studies performed to determine the incidence of anosmia and dysgeusia report heterogeneous results, ranging from 5% to 88% [8,11,12]. The most common method used to investigate the incidence of smell and taste alterations is the administration of questionnaires to patients who have tested positive for COVID-19 [13]. Other authors have collected data from patients’ records. Very few studies have employed objective olfactory tests to assess the presence of olfactory dysfunction [14]. As the correlation between subjective smell perception and the results of the olfactory tests can be low or even absent [15], relying only on patients’ reports on olfactory and gustatory dysfunctions may lead to biased results and underestimation of the real incidence of these symptoms in COVID-19 patients, also taking into consideration the fact that not all patients that present with olfactory dysfunction exhibit total anosmia. Some of them only exhibit a partial loss of smell, which, if very mild, may go unnoticed. Furthermore, only through objective measurements is it possible to quantify the loss of smell and monitor its recovery [14]. There is very little data in the literature regarding the duration of olfactory and gustatory dysfunction. Some authors report full recovery within a month from the onset of symptoms in the majority of patients, while a small percentage of patients exhibit partial recovery or no recovery [15,16,17].

The present study performed an objective evaluation, through the use of validated psychophysical olfactory tests, of the prevalence of olfactory dysfunction in a group of COVID-19 patients who had not reported smell and taste alterations during the disease. Patients were evaluated on average 131 days after their COVID-19 diagnosis.

## 2. Methods

A total of 20 subjects (11 males, 9 females) were recruited for this study. All of them had received a diagnosis of SARS-CoV-2 infection on average 131 days before the day the olfactory test was performed (median = 120, sd = 91.65, 1q = 40, 3q = 208). The mean age was 38.8. A questionnaire was administered prior to the objective evaluation, to assess whether any of the patients had detected loss of smell and/or taste.

Subsequently, the Sniffin’ Sticks Test (Burghart Medical Technology, Wedel, Germany, https://www.burghart-mt.de accessed on 9 January 2022) was performed on all subjects to objectively assess the olfactory performance. The test comprises three sub-tests, namely olfactory threshold (T), odor discrimination (D) and odor identification (I). The TDI score was then calculated for each subject [18].

### Statistical Analysis

We obtained the excess of global TDI score and of the specific scores (T, D and I) by computing the difference between the observed scores and the correspondent minimum values for normosmic individuals. As minimum scores for normosmic individuals, we used the first percentile of the distributions as tabulated by Oleszkiewicz et al. [19]. Therefore, the probability of an excess lower than 90% would be considered as indicator of some olfactory dysfunction; significance in this quantity was measured with an exact binomial test.

*p*-Values have been calculated and 5% was considered as the critical level of significance. The R: a language and environment for statistical computing (R Foundation for Statistical Computing, Vienna, Austria) was used for all analyses.

## 3. Results

Based on the answers that were provided from the questionnaire, none of the subjects reported olfactory and/or gustatory dysfunction.

**Figure 1:**
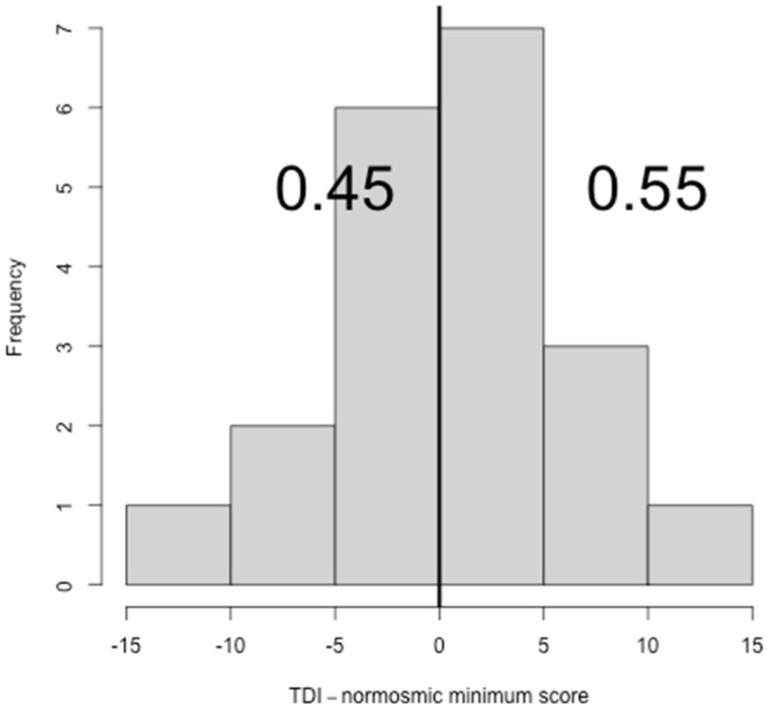
Histogram showing the difference between TDI scores and the normosmic minimum TDI score. TDI (threshold + discrimination + identification).

Based on the final TDI scores, 11 subjects scored within the normal range, therefore, showed to be normosmic (55%, CI: 32.05–76.17%), while 9 subjects showed to be hyposmic (45%, CI: 23.83–67.95%) (Figure 1). None of the subjects tested was anosmic. TDI score and both T and I sub-scores showed to be significantly different between the normosmic and the hyposmic groups (*p* < 0.000001, *p* = 0.002 and *p* = 0.002, respectively) (Table 1).

**Figure 2:**
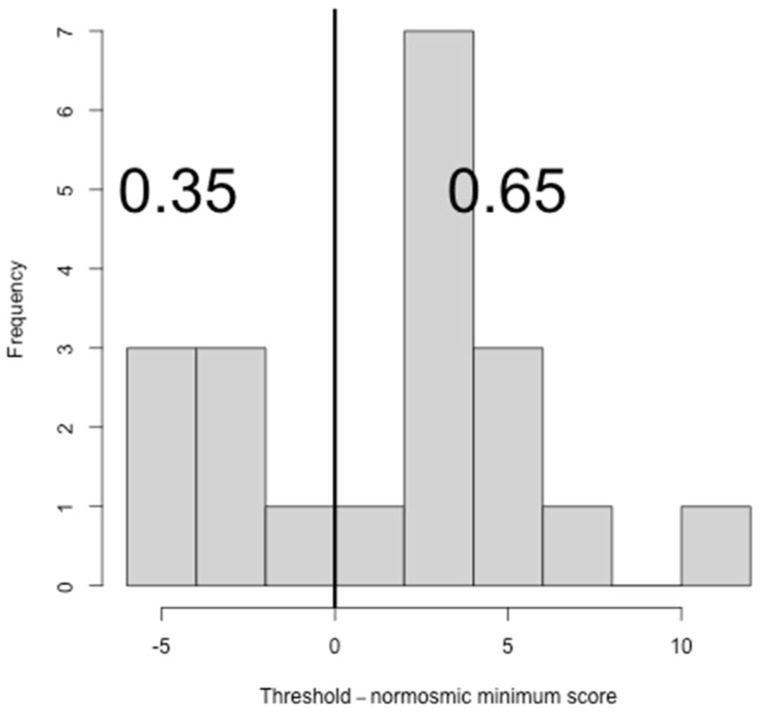
Histogram showing the difference between threshold (T) scores and the normosmic minimum threshold score.

**Figure 3:**
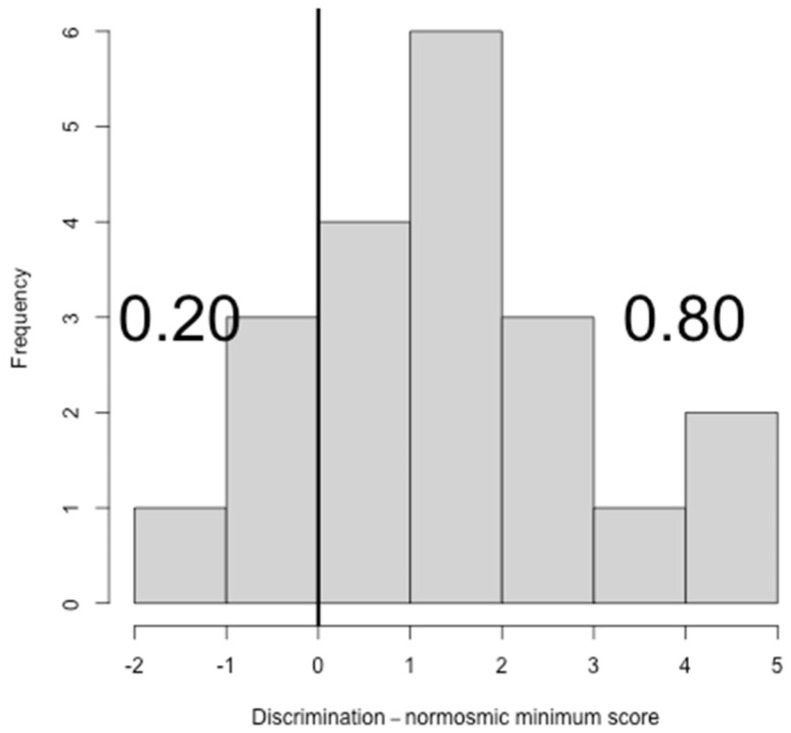
Histogram showing the difference between discrimination (D) scores and the normosmic minimum discrimination score.

**Figure 4:**
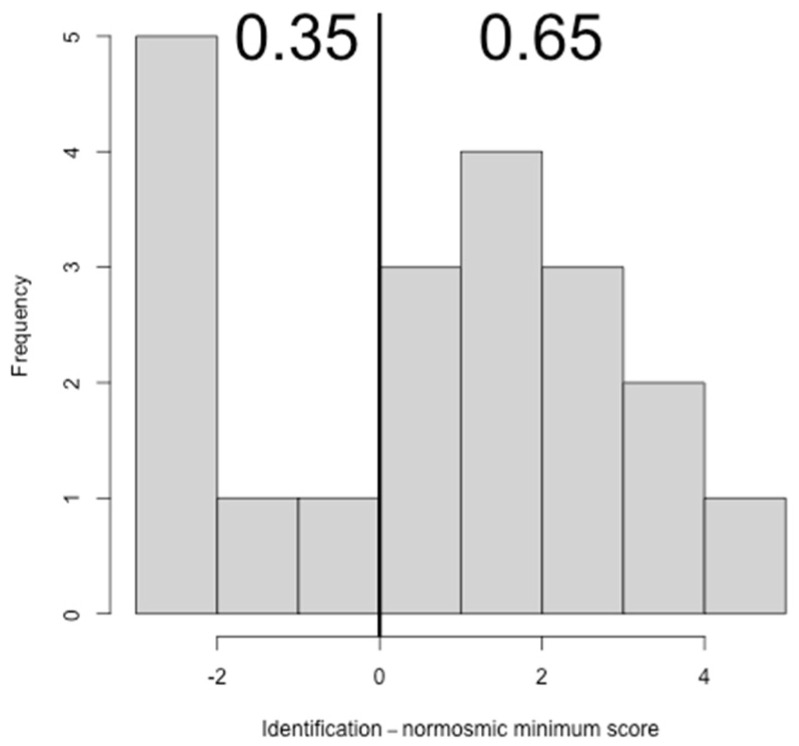
Histogram showing the difference between identification (I) scores and the normosmic minimum identification score.

Considering the results of the sub-tests, 13 subjects (65%, CI: 40.95%–83.69%) showed normal scores for T, 16 subjects (80%, CI: 55.73%–93.39%) showed normal scores for D and 13 subjects (65%, CI: 40.95%–83.69%) showed normal scores for I (Figure 2, Figure 3 and Figure 4).

## 4. Discussion

Olfactory and gustatory dysfunction have been often reported by COVID-19 patients. At this point in time, they are recognized as frequent symptoms related to SARS-CoV-2 infection; therefore, they could be used as screening questions by medical and dental professionals to help identify suspect COVID cases [20].

Studies on the prevalence of anosmia and dysgeusia report inhomogeneous findings. Taste dysfunction has shown to be present in around 71% to 88% of COVID-19 positive patients, slightly more prevalent compared to olfactory dysfunction, which has a prevalence that ranges from 68% to 85% of COVID-19 positive patients. Very often, these disturbances present concomitantly [21], suggesting that taste dysfunction in these patients is not linked to an impairment of gustation itself, but to a retronasal impairment [22].

In a study conducted by Lechien et al. on a sample of 417 COVID-19 cases, OD appeared as the first symptom in 11.8% of all cases [10]. Kaye et al. report some very interesting data collected through the COVID-19 Anosmia Reporting Tool for Clinicians, a platform established by the American Academy of Otolaryngology-Head and Neck Surgery (AAO-HNS) in order to gather data regarding olfactory symptoms. The authors analyzed the data of the first 237 entries in the platform. They report olfactory dysfunction as the first COVID-19 symptom in 27% of patients; OD was the reason the COVID-19 test was recommended in 40% of all cases [23]. These results indicate that, if recent onset of loss of smell and taste is reported by patients, they should be considered suspect COVID–19 cases and testing should be recommended.

OD and GD are not represented only by complete loss of smell and/or taste; these alterations may present themselves at different degrees, separately or concomitantly with each other and in some cases, they may be subtle and undetectable by the patient [24]. Studies performed to determine the prevalence of OD and GD in COVID-19 patients report a wide range of results. There appears to be a notable difference between results based on patients’ self-reported OD questionnaires and results that are based on objective measurement methods of olfactory performance [14,25]. A meta-analysis conducted by Pang et al. which included 19 published studies, concluded that, although not statistically significant, the prevalence of OD detected through olfactory tests was higher than the one assessed through self-reported questionnaires [24]. The authors suggest that patients may not notice smell reduction when it is very mild, especially if there are other more severe respiratory symptoms overshadowing it.

Moein et al. conducted a case-control study, in which COVID-19 positive subjects and healthy subjects underwent olfactory assessment using a validated objective test. The data they collected show a significant difference in test scoring between COVID-19 patients and healthy subjects. More than half of COVID-19 subjects presented with severe hyposmia or anosmia, while the rest of the group showed mild to moderate hyposmia. Interestingly, only 35% of the subjects had noticed and reported olfactory dysfunction [26]. Other authors that also have employed objective olfactory testing methods conclude that the prevalence of smell dysfunction is higher than the one based on patients’ reports [17]; furthermore, as reported by Vaira et al., there may be a residual olfactory deficiency even in patients that report full recovery [17]. These conclusions are in line with the results of the present study.

Little has been published regarding the duration and recovery of olfactory and gustatory alterations.

Beltrán-Corbellini et al. conducted a study on 79 COVID-19 patients and 40 controls. COVID-19 patients reported smell and test disorders lasting on average 7.5 days. Almost half of the patients recruited for their study recovered fully after 7.4 days. In some patients, smell and tasted disorders resolved after 9 days; no patient-reported persistence of OD and GD. However, the results were based on questionnaires to which the patients had to answer [27].

A multicenter study conducted by Vaira et al. concluded that severe OD and GD lasted on average 10 days; after the tenth day, smell and taste improved significantly. Almost 70% of the study group presented smell and taste disorders even 25–30 days after the onset of symptoms, but the disorders were represented by mild hyposmia and hypogeusia. The authors encountered, through objective measurement methods, mild to moderate hyposmia in almost 70% of patients that had reported full resolution of OD. They also reported residual hypogeusia in almost 30% of these patients [28]. Another smaller study showed an OD improvement after two weeks from the diagnosis [29].

Yan et al. conducted a cross-sectional study involving 1480 patients that presented symptoms similar to influenza. Out of 59 COVID-19 positive patients that responded to the survey, 40 patients reported olfactory dysfunction and 42 patients reported gustatory dysfunction. A total of 72% of patients that reported olfactory dysfunction experienced improvement 1 to 4 weeks after the onset of symptoms. Most patients reported improvement of the sense of taste contemporarily [30].

Otte et al. evaluated a group of 80 patients who had reported olfactory impairment, 8 weeks after the olfactory symptoms appeared. The authors found that almost half of the participants (45.1%) still showed to be hyposmic after 8 weeks. Not all the hyposmic subjects, although were aware of the persistence of the olfactory symptom [31].

Iannuzzi et al. evaluated 30 COVID-19 positive patients at 25 days after their COVID-19 diagnosis and at 1 month after the first evaluation, when patients no longer tested positive for the virus. The results of the Sniffin’ Sticks Test showed that there was a significant improvement from the first to the second evaluation, especially in Threshold and Discrimination values. At the first evaluation 53.3% of subjects showed to be hyposmic, while only 26.7% showed to be hyposmic at the second evaluation [32].

In general, authors conclude that smell and taste disorders resolve when other COVID-19 symptoms start to disappear; however, most studies employ subjective methods to assess the presence of OD and GD and their recovery. The results we obtained from our study demonstrate that, in some patients, recovery can be very slow and olfactory dysfunction may be present even months after its onset. Patients may be unaware of it, especially if the dysfunction is mild. It is necessary to underline that it is not possible to exclude that the hyposmic patients of the present study could have been suffering of olfactory loss before the pandemic and could be unaware of it. Nevertheless, the incidence of hyposmia in the general population is about 15% [33]. Furthermore, the subjects enrolled for this study were quite young and their history excluded other major causes of olfactory loss, a part COVID-19 infection. All in all, it seems difficult that these patients did not suffer from a mild post-viral olfactory dysfunction due to COVID-19 infection.

Efforts have been made to explain the pathophysiology of OD and GD by different authors. Rhinorrhea and nasal obstruction are not plausible pathophysiological hypotheses for explaining smell alterations, as OD is often seen in patients who do not present these symptoms [34].

Spike glycoprotein is a membrane protein that allows the virus to enter the cells of the host. The virus targets the respiratory system; it invades and replicates within the alveolar cells in the lungs and results in respiratory symptoms [1]. Similar to other coronaviruses, SARS-CoV-2 not only attacks the respiratory system but also causes disease to the gastrointestinal system, nervous system, etc. To be able to enter the cells, the novel coronavirus binds angiotensin converting enzyme 2 (ACE-2), which is an enzyme that regulates blood pressure by inhibiting the angiotensin-renin-aldosterone pathways. ACE-2 is widely distributed in the cell membranes of many organs and there is a high density of ACE-2 in the nasal and oral mucosa. The link between the spike protein and ACE-2 receptors is aided by a protease present in the surface of the target cell called TMPRSS2 [35].

One suggested hypothesis on the pathophysiology of anosmia and/or dysgeusia regards the direct damage SARS-CoV-2 may cause to the olfactory receptors present in the nasal mucosa and the gustatory receptors present in the tongue [35,36]. Olfactory and gustatory receptors bind the smell and taste molecules. The inflammation caused by the binding of the virus with the ACE-2 receptors could directly affect the activity of the receptors, thus impairing the sense of smell and taste. Furthermore, it would also reduce their availability for odorants and tastants. The ACE-2 receptors are widely distributed in the nasal mucosa and the tongue; they are also present in the epithelium of salivary glands [37].

Another advanced hypothesis regarding anosmia and dysgeusia regards the ability of the virus to cause direct damage to the olfactory neurons and neurons responsible for the sense of taste, particularly the chorda tympani which can be reached by the virus through the eustachian tube [38]. This hypothesis, however, seems unlikely given the fact that in most anosmia cases, patients recover their sense of smell within 1–2 weeks, which is a shorter time than the one required for neuronal repair [38].

Recent data provided by experiments on mice have shown that the virus does not cause direct damage to the neurons, as they do not express ACE-2 receptors. Instead, the virus most likely alters the function of the olfactory epithelium by damaging the sustentacular cells and Bowman cells [35]. The olfactory epithelium contains the cells which provide metabolic support to olfactory neurons and present similar levels of ACE-2 receptors to those of the respiratory tract. The damage caused to the epithelium decreases the trophic support that it provides to the neurons, thus causing alterations of the sense of smell [39]. The results of the present study together with those of a previous one [15], showing mainly an involvement of T, seem to confirm that OD in COVID-19 patients has an end-organ failure pathogenesis [40].

Olfactory and gustatory dysfunctions, especially if long-lasting, may impair the patients’ quality of life. However, as mentioned above, most patients recover spontaneously in a short period of time. The prescription of corticosteroid sprays to patients is not supported by the current literature because it does not seem to improve olfaction [41].

## 5. Conclusions

We conclude that self-assessed olfactory dysfunction data are not congruent with the data obtained using validated olfactory tests, such as the Sniffin’ Sticks test. Olfactory dysfunction appeared to be present even in patients who did not report smell loss. Patients often report full recovery on average 1 week from the onset of symptoms. The data we obtained show that the recovery process can be slow; OD may be present even months after the COVID-19 diagnosis, although patients may be unaware of it.

The Sniffin’ Sticks test is the best way to diagnose olfactory dysfunction in clinical practice (in the present study allowed to recognize 45% of patients with hyposmia). Nevertheless, because the duration of the complete test is rather long and necessarily requires the presence of a healthcare professional, performing only Threshold and/or Identification tests can provide significant data (in the present study, both the threshold and the identification subtests allowed to identify 35% of patients with OD). On this regard, even an identification test with 12 items could still provide significant data for diagnosing olfactory alterations, as showed by Vandersteen et al. [42], while, similarly to our study, the discrimination subtest seems to be the less useful in detecting OD.

Dentists working on the patients’ airways during dental procedures are one of those professionals most exposed to aerosolized particles. Most dental procedures involve aerosol production. Furthermore, the transmission of the virus may occur not only through aerosol, but also from contaminated instruments and various other objects in the working place [43]. Guidelines regarding the use of masks and other protective devices have been recommended to reduce the risk of infection; screening and triage protocols have been employed to detect infected patients before starting the dental treatment [44]. The results of the present study are relevant from a practical point of view. Performing only one or two of these quick Sniffin’ Sticks subtests (namely threshold and/or identification) could be an efficient screening method in clinical settings that involve high COVID-19 transmission risk, such as dental offices where professionals need to work on the airways. In this regard, COVID-19 Ag rapid test showed a sensitivity that ranges from 70 to 81%, while Reverse Transcriptase Real-time PCR, which is the gold standard for the identification of COVID-19 infection, is not user friendly and suffers of higher costs [45]. The use of a simple olfactory test could enhance the sensitivity of the former and could help the diagnosis in those asymptomatic subjects who do not have indications for a molecular COVID-19 test. Furthermore, questions regarding olfactory symptoms should be included in screening questionnaires, although, as our results showed, the answers patients provide to these questions are not as reliable as objective testing. Finally, it is worth mentioning that povidone-iodine antiseptic preparations have been shown to rapidly inactivate SARS-CoV-2 virus in vitro and that nasal and oral povidone-iodine washings have been proposed to help health care providers and especially those who work with the airways, to protect themselves during the SARS-CoV-2 pandemic [46]. Furthermore, thanks to the ability to reduce the viral load, povidone-iodine oro-nasal spray can be effective to reduce COVID-19 symptoms in the infected patients and can reduce SARS-CoV-2 transmissibility to close contacts/family members [47]

## Figures and Tables

**Table 1:** Statistical analysis.

Indicator	All Patients		
	n	Prob	*p*-Value Prob ≥ 0.9
TDI > cutoff	11	0.55	<0.001
Threshold	13	0.65	0.002
Discrimination	16	0.8	0.133
Identification	13	0.65	0.002
Total	20		

## Data Availability

The data presented in this study are available on request from the senior author (G.O.). The data are not publicly available due to privacy reasons.
